# Evaluation and comparison of ECG-gated techniques at 1.5 T for contrast enhanced MR angiography of the thoracic aorta

**DOI:** 10.1186/1532-429X-17-S1-P397

**Published:** 2015-02-03

**Authors:** Tendoh Timoh, Ruth P Lim, Mary Bruno, Gary R McNeal, Yutaka Natsuaki, Monvadi B Srichai

**Affiliations:** 1 Cardiology, Medstar Washington Hospital Center, Washington, DC, USA; 2 Radiology and Surgery, University of Melbourne, Melbourne, VIC, Australia; 3 Cardiology, Medstar Georgetown University Hospital, Washington, DC, USA; 4 Customer Solutions, Siemens Medical Solutions USA, Inc, Malvern, PA, USA; 5 Siemens Medical Solutions USA Inc., Los Angeles, CA, USA; 6 NYU Langone Medical Center, New York, NY, USA

## Background

3D T1-weighted contrast-enhanced MRA (CE-MRA) is routinely used for non-invasive evaluation of the thoracic aorta. However, competing demands of high spatial resolution and fast (breath-hold) acquisition often preclude ECG-gating, leading to motion artifact at the aortic root. Standard Cartesian-sampled ECG-gated CE-MRA acquires 1 partition per heartbeat resulting in long scan times, often exceeding breath-hold capabilities. We evaluated image quality and diagnostic capabilities of a novel ECG-gated CE-MRA utilizing alternative Cartesian k-space sampling whereby adjacent k_y_ and k_z_ points are acquired in a zigzag pattern (Z-MRA) to improve scanning efficiency and co-ordinate contrast timing with optimal cardiac phase acquisition.

## Methods

42 patients (12 females, mean 52y) were enrolled and underwent CE-MRA at 1.5T (Avanto, Siemens Healthcare) using a two-injection protocol with standard ECG-triggered CE-MRA (S-MRA) and zigzag (Z-MRA) ECG-gated CE-MRA (IPR #573: Siemens Healthcare, Germany) performed in a randomized order following 0.15mmol/kg gadolinium contrast. S-MRA parameters were: TR 2.7/TE 0.9, FA 17°, FOV 400mm. Z-MRA parameters had matched spatial resolution and FOV with other parameters: TR 2.6ms/TE 0.9ms, FA 20°, time to center (TTC) approximately 4.5s, TTC per heartbeat (k_y_=0) acquired on average 566ms post-trigger, 2-3 k_z_ loops per heartbeat (heart-rate dependent). A parallel imaging factor of 2 was used for both. Two physicians independently reviewed the images. Ten arterial segments were graded for image quality (IQ), artifacts, vascular contrast, pathology and diagnostic confidence.

## Results

1680 segments (840 x 2 readers) were evaluated. No scans were considered non-diagnostic. Average scan time was significantly longer with S-MRA compared to Z-MRA (52.4 vs. 17.9 sec, p<0.001). Overall image quality was similar for S-MRA compared to Z-MRA (Table 1). Sinus and sinotubular junction IQ and artifact scores were significantly superior for S-MRA, but beyond the ascending aorta, IQ and artifacts scores were significantly superior for Z-MRA. Vascular contrast was significantly superior at all segments for Z-MRA. Overall diagnostic confidence was significantly better for S-MRA, mainly due to difficulty discerning pathology at the aortic root.

**Table 1:** Overall comparison scores for both sequences.

Categories	Standard (S-MRA)	New (Z-MRA)	P value
Average scan time in seconds	52.4±3.5	17.9±1.4	<0.001

Diagnostic Confidence (higher better)	4.83±0.30	4.52±0.46	<0.001

Vascular contrast (higher better)	3.55±0.72	4.51±0.61	<0.001

Artifact (higher better)	3.97±0.89	3.80±1.21	<0.001

Overall Image Quality	3.86±0.88	3.85±1.20	0.867

Image Quality (annulus)	3.96±0.55	3.69±0.61	0.018

Image Quality (sinus)	3.96±0.53	3.58±0.70	0.007

Image Quality (STJ)	4.11±0.51	3.74±0.77	0.010

Image Quality (asc ao)	4.32±0.58	4.24±0.61	0.51

Image Quality (arch)	4.07±0.54	4.74±0.45	<0.001

Image Quality (desc ao)	4.46±0.52	4.88±0.36	<0.001

STJ (sinotubular junction), asc ao (ascending aorta), desc ao (descending aorta).

## Conclusions

ECG-gated Z-MRA is feasible for diagnostic evaluation of the thoracic aorta with significantly superior vascular contrast and comparably lower breath-hold times. However, motion artifact at the aortic root led to decreased diagnostic confidence at these segments for Z-MRA. Z-MRA provides a reasonable alternative to S-MRA, particularly for patients with limited breath-hold capabilities or in whom we want to limit contrast dose. Further optimization of Z-MRA k-space sampling strategies are needed to improve overall diagnostic performance.

## Funding

N/A.

